# Long Non-Coding RNA SNHG1 Regulates the Wnt/β-Catenin and PI3K/AKT/mTOR Signaling Pathways *via* EZH2 to Affect the Proliferation, Apoptosis, and Autophagy of Prostate Cancer Cell

**DOI:** 10.3389/fonc.2020.552907

**Published:** 2020-10-22

**Authors:** Junyi Chen, Fubo Wang, Huan Xu, Lingfan Xu, Dong Chen, Jialiang Wang, Sihuai Huang, Yiqun Wen, Longmin Fang

**Affiliations:** ^1^Department of Urology, The Second Affiliated Hospital of Fujian Medical University, Quanzhou, China; ^2^Department of Urology, Shanghai Changhai Hospital, Naval Medical University, Shanghai, China; ^3^Department of Urology, The First Affiliated Hospital of Anhui Medical University, Hefei, China

**Keywords:** prostate cancer, long-chain non-coding RNA, cell viability, apoptosis, autophagy

## Abstract

**Background:**

Prostate cancer (PCa) is the most common malignant cancer in western developed countries, which has seriously threatened the life style and life quality of men. Its pathogenesis and causes remain indistinct. Currently, it is found that lncRNA-SNHG1 (SNHG1) is highly expressed in multiple tumors with proto-oncogene effect, but its function and mechanism in PCa need to be further studied.

**Methods:**

The expression of SNHG1 and EZH2 was detected by RT-qPCR in the 20 pairs of PCa tissue, adjacent tissue and PCa cell lines. They were transfected with siRNA NC, SNHG1 siRNA, EZH2 siRNA, SNHG1 siRNA+empty, and SNHG1 siRNA+EZH2 overexpression. Then, MTT and colony formation assay were used to detect the proliferation and cloning ability of PCa cells LNCaP and PC3. Transwell and flow cytometry were used to measure cell migration and invasion ability and apoptosis level respectively. Immunofluorescence was used to detect the LC3 spot formation. Western blot was used to detect the expression of the autophagy-related proteins, and PI3K/AKT/mTOR and Wnt/β-catenin signaling pathway related proteins. Finally, *in vivo* nude mice tumorigenesis experiment to explore the effect of SNHG1 expression on PCa.

**Results:**

We found that SNHG1 and EZH2 were up-regulated in PCa tissue and cells. The expression of SNHG1 and EZH2 was positively correlated. RNA pull down and RNA IP assay further confirmed that SNHG1 bound to EZH2. The proliferation, colony formation, migration and invasion of LNCaP and PC3 cells were significantly reduced with the interference with SNHG1or EZH2 compared with the control group. The related proteins of Wnt/β-catenin and PI3K/AKT/mTOR signaling pathway were significantly reduced after the interference with SNHG1 or EZH2; after simultaneous interference with SNHG1 and overexpression of EZH2, the functional effects on LNCaP and PC3 cells interfered with SNHG1 were reversed. These results were also confirmed *in vivo* nude mice tumor formation experiments.

**Conclusions:**

This study reveals that lncRNA-SNHG1 regulates Wnt/β-catenin and PI3K/AKT/mTOR signaling pathways via EZH2 gene to affect proliferation, apoptosis and autophagy of PCa cells. This experiment provides ideas and experimental basis for the improvement and treatment of PCa.

## Introduction

Prostate cancer (PCa) is the most widespread malignant cancer in western developed countries (1). For example, it is the second pervasive tumor among men in the United States (2). A study conducted by the American Cancer Society in 2012 showed that PCa ranks first in new tumors among all American men in one year (29%), and its incidence varies greatly among different races, which is relatively low in China compared with western developed countries (3). However, as China begins to enter an aging society, the impact of PCa on the lifestyle and life quality of Chinese men has become increasingly prominent (4). Moreover, the incidence of PCa has been on the rise in recent years due to the changes in diet and lifestyle and the improvement in diagnostic methods. Many patients are already in the advanced stage when diagnosed because of the insidious symptoms. At that time, the therapeutic effect of PCa is usually poor (5). Therefore, early diagnosis and intervention of PCa will help to reduce its harm to patients and improve the therapeutic effect. However, even with the in-depth study of PCa, the pathogenesis and causes of PCa have not been fully understood (6). This situation has led a large number of scholars to continuously search for molecular indicators for early diagnosis of PCa, molecular targets for PCa treatment and molecular markers for the prognosis of PCa.

Long non-coding RNAs (lncRNAs) are endogenous RNAs with a length of more than 200 nucleotides, which are completely absent or have the ability to encode small peptides (7–9). LncRNAs can participate in the pathological process of cancer through various mechanisms from transcription level to post-transcription level (10, 11). Recent studies revealed that some lncRNAs can regulate and take part in the pathological development of some PCa (12, 13). For instance, lncRNA-SPRY4-IT1 can contribute to the proliferation of human PCa cell Du145 and is considered as a PCa oncogene (14). And some lncRNAs can interact with NF-κB signaling pathway to form stable complexes, which can block the phosphorylation of P52 protein to inhibit the metastasis of PCa cells (15).

Latest reports indicated that lncRNA nucleolar small molecule RNA host gene 1 (SNHG1) is located on chromosome 11 and is abnormally overexpressed in a variety of tumor tissue and can accelerate the cell proliferation in lung cancer (16), liver cancer (17), gastric cancer (18), esophageal cancer (19) and PCa cells (20). It is closely associated with the adverse prognosis of these tumors. Moreover, SNHG1 is up-regulated in lung cancer tissue and is associated with poor prognosis (21). Meanwhile, SNHG1 can be detected by using the urine of patients to help patients avoid invasive examination of prostate. In addition, studies have shown that SNHG1, as an oncogene, can promote the proliferation and invasion of colorectal cancer cells and inhibit the apoptosis (22). Although the detection of SNHG1 in urine has been approved by FDA, its function and mechanism remain unclear. This study explored the effect of SNHG1 on the proliferation, apoptosis and autophagy of PCa cells and its mechanism, providing a new theoretical basis for the treatment of PCa.

## Materials and Methods

### Collection and Processing of Clinical Specimens

The tumor tissue and adjacent normal tissue of 20 patients with PCa admitted to the Second Affiliated Hospital of Fujian Medical University from June 2016 to December 2017 were collected. And their basic information was recorded, including age, gender, blood pressure, tumor stage, distal metastasis (with or without) and slymphatic metastasis (with or without). The postoperative pathological stages of all the patients were determined by the joint review of three pathologists. The tumor tissue was divided into two parts. One part was stored with RNA preservation solution, and the other part was stored with liquid nitrogen after rinsed with cold phosphate buffer (PB) treated with diethyl pyrocarbonate (DEPE) to remove blood. All the patients had not received chemotherapy or radiotherapy before the operation, with PCa as the primary lesion confirmed by the pathological examination and without history of major diseases. The patients signed the informed consent.

### Cell Culture and Cell Transfection

Human PCa cell lines LNCaP, PC-3, DU-145 and normal prostate epithelial cell RWPE-1 were purchased from the Cell Center of the Institute of Basic Medical Sciences, Chinese Academy of Medical Sciences. Cells were cultured in the F12K medium (Gibco, USA) added with 10% fetal bovine serum (Gibco, USA) at 37°C in a humidified 5% CO_2_ incubator. The SNHG1 siRNA and EZH2 siRNAs were designed and synthesized by Ribobio (Guangzhou, China). Full-length cDNAs of human EZH2 were synthesized and cloned into the expression vector pcDNA3.1 (Ribobio, China). The plasmid and siRNAs were transfected into LNCaP and PC-3 cells using Lipofectamine 3000 according to the manufacturer’s instructions. The cells are divided into 5 groups: siRNA NC (NC), SNHG1 siRNAs (si-SNHG1), EZH2 siRNAs (si-EZH2), SNHG1 siRNAs + blank vector (si-SNHG1+con), si-SNHG1+EZH2 overexpression vector (si-SNHG1+EZH2).

### MTT Assay

The cells in logarithmic growth phase were digested and counted. Cells after transfection were cultured in 96-well plates with 2,000 cells/well, and five parallel wells were set for each sample. Finally, a total of four 96-well plates were set. After the cells were transfected 1d, 2d, and 3d, each well was added with MTT solution and incubated at 37°C for another 4 h. The medium was then sucked out, and 150 μl DMSO was added into each well. The absorbance of the plate (490 nm) was detected by a microplate reader.

### Transwell Assay

The cells in the logarithmic growth phase were digested and counted, and were cultured in the upper transwell chamber, with 50,000 cells/well and 200 µl serum-free medium. Subsequently, 600 μl medium containing 20% FBS was added to the lower chamber. It was incubated at 37°C with 5% CO_2_ for 17 h. The transwell chamber was fixed with methanol for 15 min and stained with 0.5% crystal violet for 20 min. Ultimately, the cells in the upper chamber were removed, and 10 fields were randomly selected to take photos of the cells on the lower surface of the polycarbonate membrane, and the cells were counted and statistically analyzed.

### Flow Cytometry

The cells after transfection were digested and collected. The 5–10 × 10^5^/ml cells were added 195 μl Annexin V-FITC binding solution and were resuspended. Then they were added with 5 μl Annexin V-FITC reagent, gently mixed, and placed in the dark at room temperature for 10 min. Subsequently, 10 μl propidium Iodide (PI) was added, mixed gently, and placed in the dark at room temperature for 10 min. Then 200 μl Annexin V-FITC binding solution was added and cells were gently resuspended. The sample was eventually detected by the flow cytometry, in which green fluorescence was shown after binding Annexin V-FITC and red fluorescence was shown after binding PI.

### Immunofluorescence

Cells of the same generations were inoculated on the cover slip. After 48 h of transfection, the cover slip was rinsed with PBS for 3 times (10 min each time). After that, it was fixed with 4% paraformaldehyde for 15 min, rinsed with PBS, treated with 0.5% Triton X-100 for 10 min, and rinsed with PBS again. Then,.100 mL/L bovine serum albumin was added to seal for l h. LC3 antibody (Abcam, ab192890) with a concentration of 1:5,000 was added overnight at 4°C. After PBS rinsing, human FITC-labeled immunofluorescent antibody was added at 37°C for l h. Then the cover glass was rinsed with PBS and sealed with glycerin after washed with distilled water.

### RNA Pull-Down

The cells in the 6 cm dish with cell confluence of 90% were scraped with a cell scraper, and the cell scraper and the dish were rinsed with 1 mL pre-cooled PBS. The suspension was collected and placed in the EP tube. The above operation were all completed on ice. The cells were centrifuged at 12,000 × g 4 °C for 30 s to obtain cell precipitate and the supernatant was aspirated. A total of 300 μl cell lysate, protease inhibitor, and phosphatase inhibitor were added to lyse the cells at 4°C for 1 h. The cell lysate was centrifuged at 12,000×g 4°C for 10 min, and the lysate supernatant was collected in DEPC-treated EP tube without RNase. Biotin labeled 3’UTR probe (about 400 ng), yeast RNA (100 g/L, 1.5 μl), RNase inhibitor and heparin were added to the lysis supernatant and incubated at 4°C for 3 h. A total of 20 μl streptavidin-labeled beads were placed in an RNase-free EP tube, rinsed with 500 μl cell lysate, and then centrifuged at 500 × g for 5 min at 4°C for later use after supernatant aspirated. After the incubation of the probe with the lysate for 3 h followed by centrifugation at 12,000 × g for 5 s at 4°C, the mixed solution was transferred to the previous beads for 3 h incubation at 4°C, and them rinsed with 750 μl of cell lysate to remove the non-specific proteins bound with the beads. Centrifuged at 500 × g for 5 min at 4°C and aspirated the supernatant. A total of 20 μl 2 × loading buffer for denaturation at 95°C for 5 min followed by centrifugation for 3 min at 12,000 r/min after the beads were rinsed for 4 times for western blotting detection.

### RNA Binding Protein Immunoprecipitation (RIP)

The activated cells were collected and rinsed twice with PBS. A total of 10 ml of PBS and formaldehyde with a final concentration of 0.01% for 15 min cell cross-linking. Then 1.4 ml of 2 mol/L glycine was added and mixed for 5 min followed by centrifugation 1,500 r/min for 5 min. The cells were washed twice with PBS with the supernatant aspirated, and then, they were lysed using RIPA.

The cell lysate was divided into two groups incubated at 4°C overnight. In the experimental group, 4 μg TNF-α antibody (Abcam) was added, while in the control group, 4 μg normal rabbit IgG was added. Then 20 μL of Protein A resin was added and incubated at 4°C for 1 h followed by low-speed centrifugation and the supernatant aspirated. The Protein A resin was rinsed with PBS for 4 times, with 50 μL of PBS for resuspension, and then divided into 2 parts after mixing. One part was used for western blotting, while the other part was used for RNA extraction with TRIzol reagent; RNA was reverse transcribed into cDNA, and the mRNA level was detected by RT-qPCR.

### RNA Fluorescence *In Situ* Hybridization (FISH)

The FISH kit was purchased from RiboBio (Guangzhou, China), and the experiment was performed according to the instructions. The LNCaP and PC-3 cells were inoculated and fixed with 4% paraformaldehyde, and treated with 0.5% Triton in PBS followed by pre-hybridization. They were then hybridized at 5 mM SNHG1 probe concentration overnight in the dark. The next day, the cells were counterstained with DAPI and imaged.

### Quantitative Reverse Transcription-Polymerase Chain Reaction (RT-qPCR)

Total RNA was isolated from LNCaP, PC-3, DU-145, normal prostate epithelial cell line RWPE-1 and the animal tumor tissue using Trizol reagent (Invitrogen) according to the instructions. A total of 500 ng of RNA was reverse transcribed into cDNA using cDNA transcription kit (ABI). Transcription was subsequently performed at 16°C for 30 min, followed by incubation at 42°C for 30 min and inactivation of the enzyme at 85°C for 5 min. Rapid quantitative PCR was performed using SYBRH Select Master Mix (Invitrogen). Instead, transcription was performed using the following parameters: 16°C, 30 min; 42°C, 30 min; 84°C, 5 min. The RT-qPCR was performed using the following parameters: 95°C for 2 min, followed by 40 cycles of 95°C, 15 s, and 60°C. All results were standardized by GAPDH expression. Quantitative analysis was used 2^-ΔΔCt^ method. The primer sequences used were as follows: SNHG1, 5’-AGCATCCACGAGCAAGAGAC-3’ and 5’-GATGCTACTAGTGTGGCGGG-3’; EZH2, 5’-GAAGCTGAGATGAGCCTAT-3’ and 5’-GACAACTGTGAAGCCAGGTT-3’; GAPDH, 5’-AACGATTTGGTTATTG-3’ and 5’-GGAAGATGTGGTATT-3’.

### Colony Formation Assay

Human PCa cell lines LNCaP and PC-3 and normal prostate epithelial cell line RWPE-1 were digested with pancreatin followed by centrifugation at 1,000 r/min for 3 min with a centrifugation radius of 12 cm, and supernatant was aspirated. A total of sterile PBS solution was added, the cells were dispersed and centrifuged again. After repeated rinsing for 3 times, the culture medium was added to prepare single-cell suspension., The concentration of PCa cells was measured and adjusted to 1,000 cells/ml. Single-cell suspension was evenly inoculated in sterile 6-well plates for colony formation. The culture medium was changed every 3 d, and the tumor cell colony formation was observed after 14 d. The experiment was repeated three times.

### Western Blot

Total protein in the cell lines and the animal tissue were extracted in accordance with the manufacturer’s procedure. All steps were performed on ice to minimize degradation. The extracted total protein concentration was determined by BCA protein assay kit (Bio-rad). The electrophoresis total protein was heated to 100°C and incubated for 5 min, followed by SDS- polyacrylamide gel electrophoresis (120 v, 100 min). The protein separated from SDS gel was then transferred to the PVDF membrane (300 mA, 80 min). After the membrane transfer was completed, the target band was sealed with 5% TBS. The membrane was incubated with an rabbit monoclonal antibody EZH2 (Abcam, ab191080), a rabbit monoclonal antibody LC3-II (Abcam, ab192890), a rabbit monoclonal antibody p62 (Abcam, ab56416), a rabbit monoclonal antibody Beclin-1 (Abcam, ab210498), a rabbit monoclonal antibody bcl-2 (Abcam, ab32124), a rabbit monoclonal antibody Wnt1 (Abcam, ab15251), a rabbit monoclonal antibody β-catenin (Abcam, ab32572), a rabbit monoclonal antibody c-myc (Abcam, ab32072), a rabbit monoclonal antibody Cyclin D1 (Abcam, ab16663), a rabbit monoclonal antibody p-PI3K (Abcam, ab32089), a rabbit monoclonal antibody p-AKT (Abcam, ab8805), rabbit monoclonal antibody p-mTOR (Abcam, ab109268) and rabbit polyclonal antibody GADPH (Abcam, ab9485) with a dilution of 1:1,000 at 4°C overnight. The next day, it was rinsed three times. Subsequently, the membrane was incubated with horseradish peroxidase (HRP) and conjugated polyclonal goat anti-rabbit IgG (Abcam, ab6721) secondary antibody (1:10,000) at room temperature for 1 h, then rinsed several times. The membrane with an enhanced chemiluminescence performance system was imaged with X-ray film. The image was then quantified with Image J(NIH).

### Mouse Xenograft Experiments

Twelve BALB/c nude mice were raised at constant temperature (25–27°C), constant humidity (25–50%) and specific pathogen-free. PC-3 cells in logarithmic growth phase were digested with pancreatin to prepare single-cell suspension, centrifuged at 1 000 r/min for 10 min to collect precipitate, rinsed with PBS twice to prepare cell suspension with a density of 1×10^7^ cells/mL. Their axillary skin was disinfected, and 200 μL PC-3 cell suspension were aspirated with sterile trocar to inoculate subcutaneously in the axillary. Ten days after the inoculation, the tumor-bearing nude mice were numbered and randomly divided into 3 groups, NC, si-SNHG1, si-SNHG1+EZH2 groups, with 3 mice in each group. Reagents in each group were dissolved in 200 μL PBS and injected every 2 d for 5 times. The tumor size and the weight of mice were measured every week. The tumor volume is calculated by the formula: Tumor volume = (tumor lengh × tumor width × tumor width)/2. The nude mice bearing tumors were sacrificed after 28 d, the xenografts were taken out and weighed and the tumor size was measured. One part of the tumor specimen was fixed with 4% paraformaldehyde, while the other part was quickly stored in liquid nitrogen.

### Immunohistochemistry (IHC)

Tumor specimens were collected fixed in 4% paraformaldehyde for 48 h, dehydrated, embedded in paraffin for section (size: 0.4 µm). The specimens were then dewaxed and hydrated with gradient ethanol. Subsequently, the sections were cooled at room temperature for 30 min, blocked with 3% hydrogen peroxide for 15 min, and incubated with primary anti-Ki67 (1:500 dilution, Abcam, ab15580) for 2 h at room temperature. After rinsed with TBST, the specimens were treated with HRP-labeled anti-goat IgG secondary antibody for 2 h at room temperature. And the cell nuclei were stained with hematoxylin.

### Statistical Analysis

The data obtained from the above experiments were statistically analyzed by SPSS 22.0 software. The measurement data were expressed by mean ± standard deviation (SD). T test was used to compare the differences between the two groups. One-way ANOVA was used to compare the differences among the three groups. Each experiment was repeated at least 3 times. P < 0.05 was considered statistically significant.

## Results

### High Expression of SNHG1 and EZH2 in PCa Tissues and Cells

**Figure 1:**
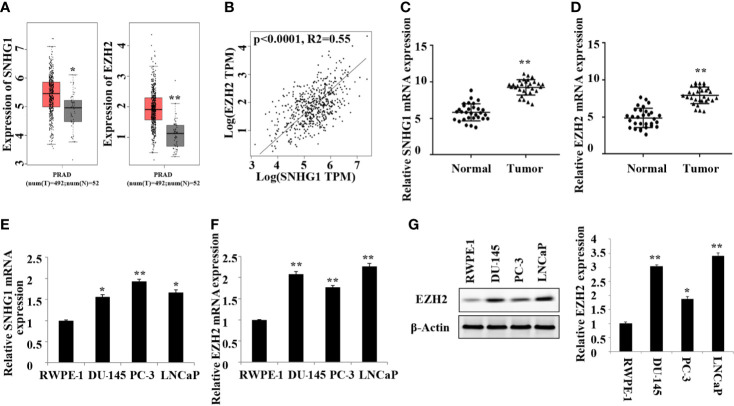
High expression of SNHG1 and EZH2 in PCa tissue and cells. **(A)** TCGA database showed that SNHG1 and EZH2 were highly expressed in PCa tissue; **(B)** The correlation analysis between SNHG1 and EZH2; **(C)** RT-qPCR to detect the expression of SNHG1 in PCa tissue and adjacent normal tissue; **(D)** RT-qPCR to detect the expression of EZH2 in PCa tissue and adjacent normal tissue; **P < 0.01 vs. the Normal group. **(E)** RT-qPCR to detect the SNHG1 expression in LNCaP, PC-3, DU-145 and RWPE-1; **(F)** RT-qPCR to detect the EZH2 expression in LNCaP, PC- 3, DU-145 and RWPE-1; **(G)** Western blot to detect the expression of EZH2 protein in PCa cell lines. *P < 0.05 compared with RWPE-1 group; **P < 0.01 vs. RWPE-1 group.

In this study, we first detected the expression of SNHG1 and EZH2 in 20 pairs of PCa tissue and adjacent normal tissue by RT-qPCR. As shown in **Figure 1A**, TCGA database analysis showed that SNHG1 and EZH2 were highly expressed in prostate cancer tissue. The results of correlation analysis showed a positive correlation between SNHG1 and EZH2 expression (**Figure 1B**). Further, as shown in **Figures 1C, D**, the mRNA expression of SNHG1 and EZH2 in PCa tissue was significantly increased compared with the adjacent normal tissue. In addition, the expression of SNHG1 and EZH2 was further confirmed in the expression of LNCaP, PC-3, and DU-145. The expression of SNHG1 and EZH2 in LNCaP, PC-3 and DU-145 cells was significantly increased compared with RWPE-1 (**Figures 1E, F**). Western blot results also confirmed a significant up-regulation of EZH2 protein expression in LNCaP, PC-3 and DU-145 cells (**Figure 1G**). These results indicated that SNHG1 and EZH2 were involved in the occurrence and development of PCa. SNHG1 has the highest expression in PC-3, and EZH2 has the highest expression in LNCaP. Therefore, PC-3 and LNCaP were used for subsequent experiments.

### SNHG1 Regulates the Proliferation of PCa Cells by Targeting EZH2

**Figure 2:**
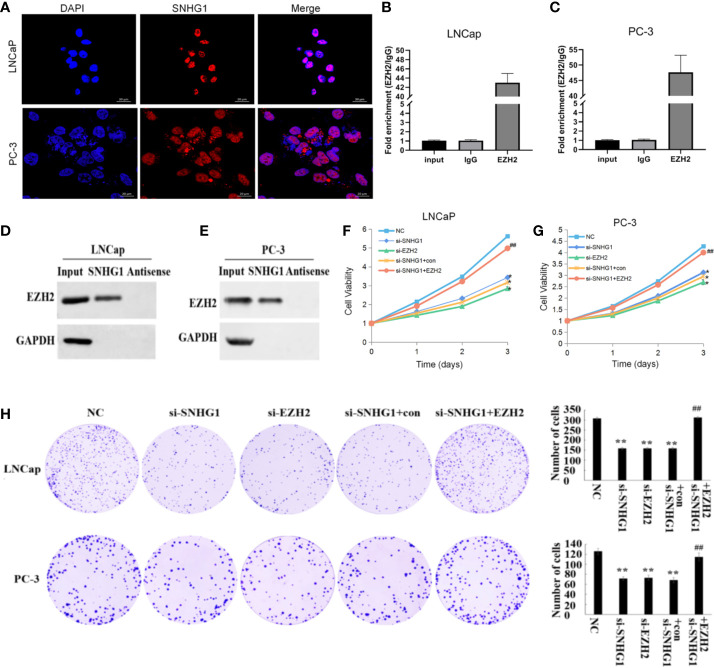
Regulatory effect of SNHG1 on proliferation of PCa cells SNHG1 by targeting EZH2. **(A)** FISH showed the subcellular location of SNHG1 in LNCaP and PC3 cells (red), and the nuclei were stained with DAPI (blue). **(B, C)** RNA Pull-down assay forward detected the binding of SNHG1 and EZH2 in LNCaP and PC3 cells; **(D, E)** RNA IP reverse verification of the binding of SNHG1 and EZH2 in LNCaP and PC3 cells; **(F, G)** MTT assay interfered with SNHG1, EZH2 down-regulated or interfered with SNHG1 while overexpressing EZH2, affecting the proliferation in LNCaP and PC3 cells; **(H)** Colony formation assay detected LNCaP and PC3 cells transfected with SNHG1 siRNA, EZH2 siRNA or SNHG1 siRNA+EZH2 after cell colony formation. *P < 0.05 and **P < 0.01 vs. NC group; ^##^P < 0.01 vs. si-SNHG1 + con group.

The subcellular localization of lncRNA is closely related to its biological function. We confirmed that SNHG1 was mainly in the nuclei of LNCaP and PC3 cells detected by FISH (**Figure 2A**). In addition, the results of RNA Pull-down (**Figures 2B, C**) and RIP (**Figures 2D, E**) showed that SNHG1 and EZH2 proteins interacted and could target each other. To further study the effect of SNHG1 and EZH2 on the proliferation of PCa cells, we chose to interfere with the expression of SNHG1 or EZH2 in LNCaP and PC3 cells. The results of MTT (**Figures 2F, G**) and colony formation assay (**Figure 2H**) showed that interference with the expression of SNHG1 and EZH2 could inhibit the proliferation and colony formation of LNCaP and PC3 cells. After interfering with the expression of SNHG1 and overexpressing EZH2, it could eliminate the inhibitory effect of SNHG1 silencing on the proliferation and cloning ability of LNCaP and PC3 cells. These experimental results confirmed that SNHG1 regulated the proliferation of PCa cells by targeting EZH2.

### SNHG1 Regulates the Apoptosis of PCa Cells by Targeting EZH2

**Figure 3:**
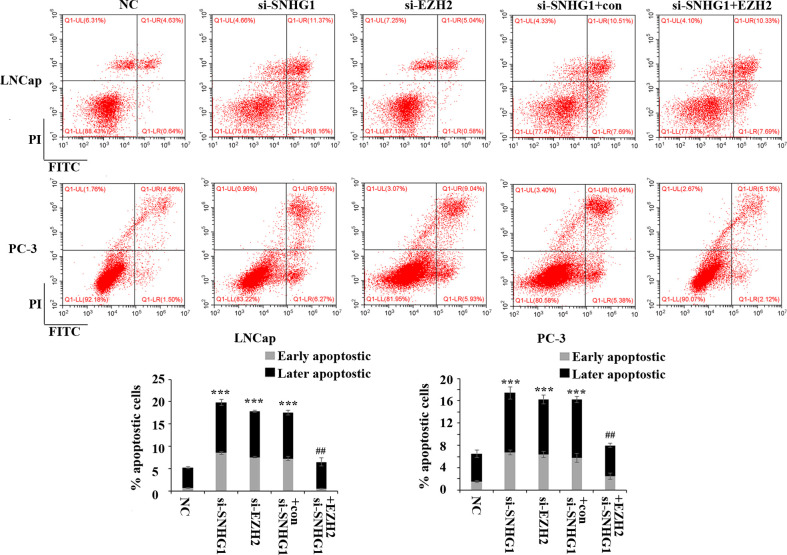
Regulatory effect of SNHG1 on the apoptosis of PCa cells by targeting EZH2. Flow cytometry was used to detect the apoptosis of LNCaP and PC3 cells transfected with NC, si-SNHG1, si-EZH2, si-SNHG1+con, si-SNHG1+EZH2. ***P < 0.001 vs. NC group; ^##^P < 0.01 vs. si-SNHG1 + con group.

In order to evaluate the effect of SNHG1 and EZH2 expression on the apoptosis of PCa cells, we performed flow cytometry in LNCaP and PC3 cells. The results showed (**Figure 3**) that down-regulating the expression of SNHG1 and EZH2 promoted the apoptosis of LNCaP and PC3 cells. Co-transfection of si-SNHG1+EZH2 reversed the promotion of apoptosis of LNCaP and PC3 cell by SNHG1 silencing.

### SNHG1 Regulates the Migration and Invasion of PCa Cells by Targeting EZH2

**Figure 4:**
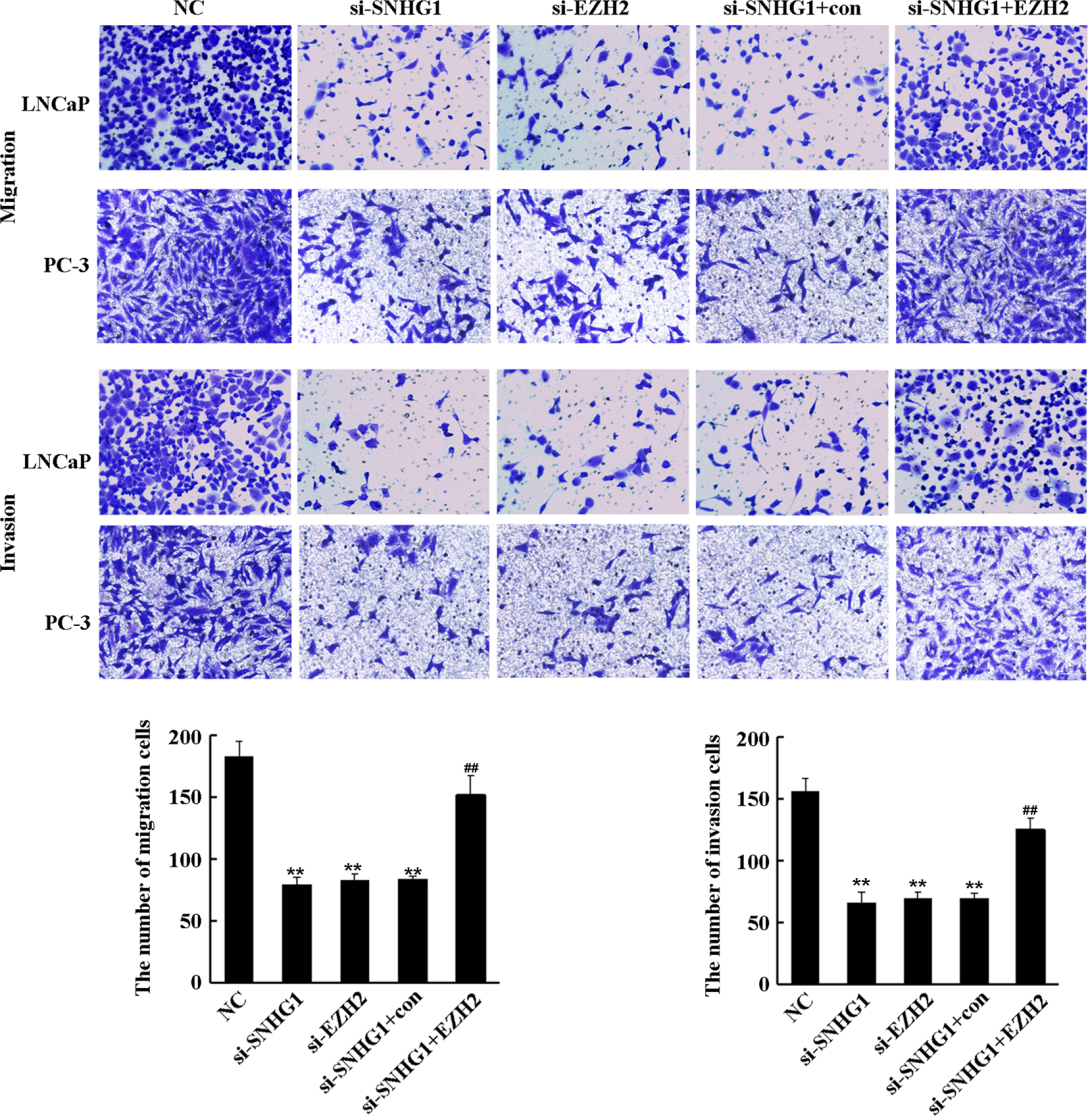
Regulatory effect of SNHG1 on the migration and invasion of PCa cells by targeting EZH2. Transwell assay detected thel migration and invasion of LNCaP and PC3 cells transfected with NC, si-SNHG1, si-EZH2, si-SNHG1+con, si-SNHG1+EZH2. **P < 0.01 vs. NC group; ^##^P < 0.01 vs. si-SNHG1 + con group.

To further evaluate the effect of SNHG1 and EZH2 expression on the migration and invasion of PCa cells, we performed Transwell assay in LNCaP and PC3 cells. As shown in **Figure 4**, the down-regulation of SNHG1 and EZH2 expression inhibited the migration and invasion of LNCaP and PC3 cells. Interference with SNHG1 and simultaneous overexpression of EZH2 reversed the inhibition of migration and invasion of LNCaP and PC3 cells by SNHG1 silencing.

### SNHG1 Binding EZH2 Inhibits Autophagy Pathway in LNCap and PC-3 Cells

**Figure 5:**
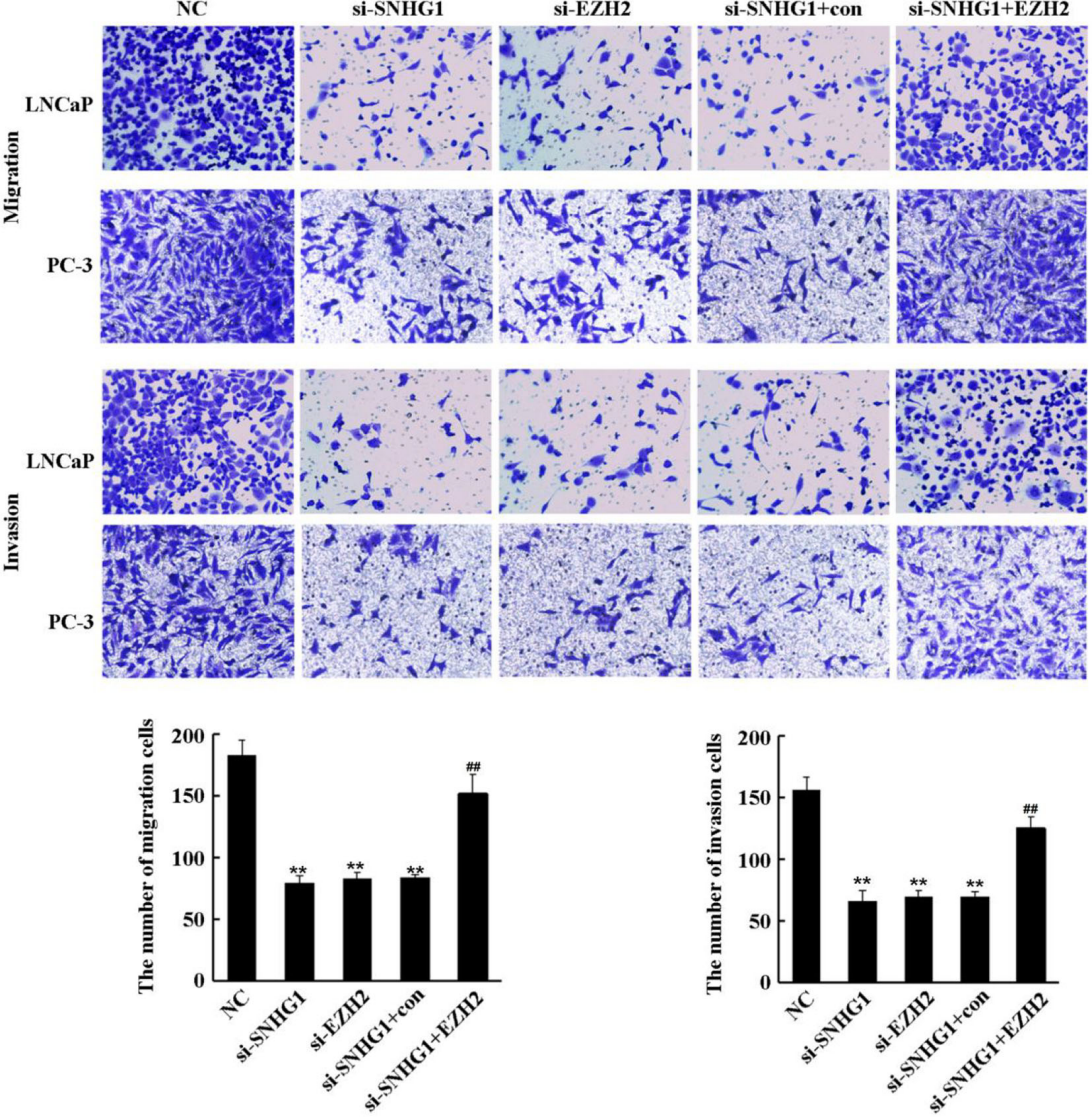
Effect of SNHG1 binding EZH2 on autophagy in LNCap and PC-3 cells. **(A–B)** Immunofluorescence staining detected the formation of the LC3 spots with the interference of SNHG1 in LNCaP and PC3 cells and the down-regulation of EZH2 or the interference of SNHG1 and simultaneous overexpression of EZH2. **(C)** Western blot detected the expression level of autophagy-related proteins LC3-II, Beclin-1 and P62 protein in LNCaP and PC3 cells transfected with NC, si-SNHG1, si-EZH2, si-SNHG1+con, si-SNHG1+EZH2.

To detect whether autophagy is involved in the function of SNHG1 on PCa cells, we used immunofluorescence staining and Western Blot to detect the expression of autophagy-related proteins in the cells. As shown in **Figures 5A, B**, compared with the NC group, the number of LC3 spots formed in LNCaP and PC-3 cells significantly increased after interference with the expression of SNHG1 or EZH2. However, the number of LC3 spots formed in the cells was significantly reduced after simultaneous interference with SNHG1 and overexpression of EZH2. Western blot showed (**Figure 5C**) that after down-regulating the expression of SNHG1 or EZH2, the protein expression of LC3-II and Beclin-1 in LNCaP and PC-3 cells was significantly increased, while the protein expression of p62 was significantly reduced. After transfection with si-SNHG1+EZH2, the protein expression of LC3-II and Beclin-1 in the cells was significantly reduced, while the protein expression of p62 was significantly increased. These results confirmed that the interference with SNHG1 expression promoted the autophagy in LNCaP and PC-3 cells, that is, enhanced the self-degradation ability of the cancer cells.

### SNHG1 Binding EZH2 Activates PI3K/AKT/mTOR and Wnt/β-Catenin Signaling Pathways in LNCap and PC-3 Cells

**Figure 6:**
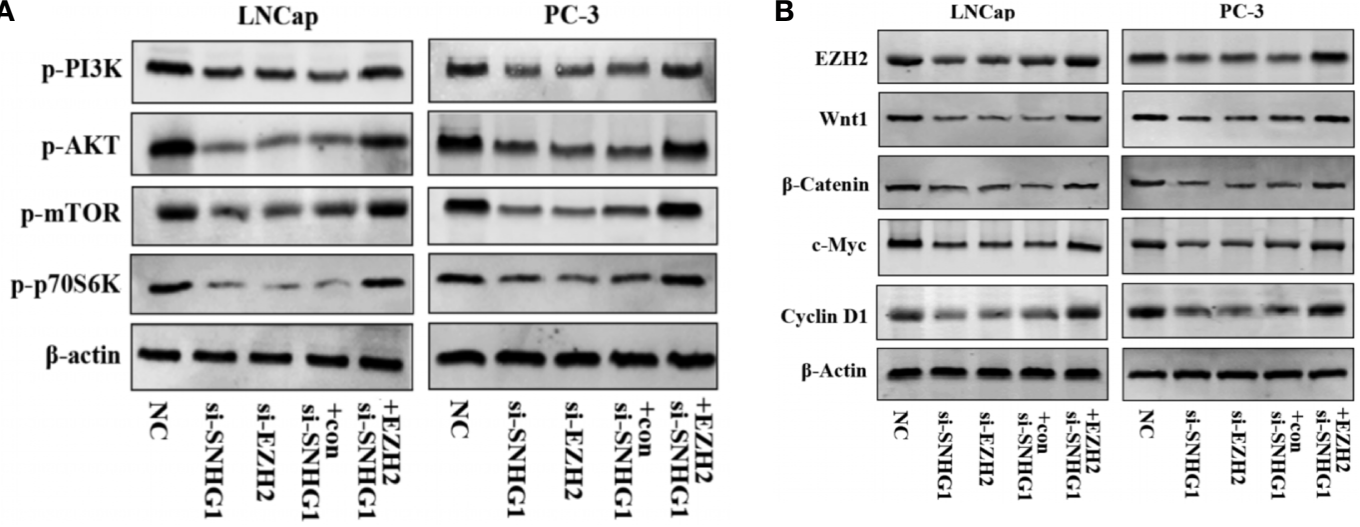
SNHG1 binding EZH2 activates PI3K/AKT/mTOR and Wnt/β-catenin signaling pathways in LNCap and PC-3 cells. **(A)** Western blot was used to detect the protein expression levels of PI3K/AKT/mTOR signaling pathway-related proteins p-PI3K, p-AKT, p-mTOR and p-p70S6K in LNCaP and PC3 cells transfected with NC, si-SNHG1, si-EZH2, si-SNHG1+con, si-SNHG1+EZH2. **(B)** Western blot was used to detect the expression levels of Wnt/β-catenin signaling pathway related proteins Wnt1, β-catenin, c-myc and Cyclin D1 in LNCaP and PC3 cells transfected with NC, si-SNHG1, si-EZH2, si-SNHG1+con and si-SNHG1+EZH2.

We further explored the molecular mechanism of SNHG1 regulating PCa cell function changes. Western blot results showed that the PI3K/AKT/mTOR signaling pathway proteins p-PI3K, p-AKT, p-mTOR, and p-p70S6K were significantly down-regulated with interference with the expression of SNHG1 or EZH2 in LNCaP and PC3 cells. And the protein expression was reversed with simultaneous interference with SNHG1 and overexpression of EZH2 (**Figure 6A**). This result confirmed that interference with SNHG1 promoted the autophagy and apotosis of tumor cells by inhibiting PI3K/AKT/mTOR signaling pathway. In addition, we found that the expression of Wnt/β-catenin pathway related proteins Wnt1, β-catenin, c-myc, and Cyclin D1 in LNCaP and PC3 cells were significantly reduced with the interference with the expression of SNHG1 or EZH2. However, the protein expression of EZH2, Wnt1, β-catenin, c-myc, and Cyclin D1 in the cells transfected with si-SNHG1+EZH2 was significantly increased (**Figure 6B**). It confirmed that Wnt/β-catenin was involved in the regulation of PCa cell function by SNHG1.

### SNHG1 Affects the Growth of Subcutaneous Tumors in Nude Mice by Regulating EZH2 Gene

**Figure 7:**
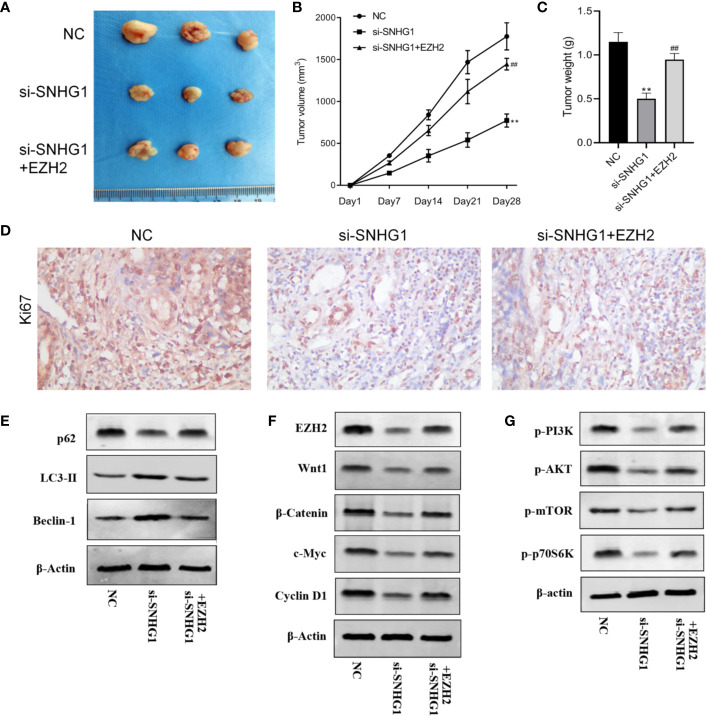
Effect of SNHG1 on the growth of subcutaneous tumors in nude mice by regulating EZH2. **(A)** Images of xenograft tumor sizes of each group. **(B)** The tumor volume growth curves after the injection in each group. **(C)** The tumor weight after the injection in each group. **(D)** Ki67 immunostaining of the animal tumor specimens from three groups. **(E)** Western blot to detect autophagy-related protein LC3-II, Beclin-1 and P62 protein expression levels in the animal tumor tissue transfected with NC, si-SNHG1, and si-SNHG1 + EZH2. **(F)** Western blot to detect Wnt/β-catenin signaling pathway-related protein Wnt1, β-catenin, c-myc and cyclin D1 protein expression levels. **(G)** Western blot to detect PI3K/AKT/mTOR signaling pathway-related protein p-PI3K, p-AKT, p-mTOR and p-p70S6K protein expression levels. **P < 0.01 vs. NC group; ^##^P < 0.01 vs. si-SNHG1 + con group.

Finally, in order to study the effect of SNHG1 on tumor growth *in vivo*, we injected PC-3 cells subcutaneously into the nude mice. The results showed that compared with the NC group, the tumor volume and tumor weight decreased gradually with SNHG1 interference. However, the tumor volume and tumor increased with simultaneous interference with SNHG1 and overexpression of EZH2, (**Figures 7A–C**). The IHC results (**Figure 7D**) also confirmed that the positive expression of Ki67 in the tumor tissue decreased after interference with SNHG1, while the positive expression of Ki67 increased after interference with SNHG1 and overexpression of EZH2. Western Blot results showed that the protein expression of LC3-II and Beclin-1 in the tumor tissue increased significantly, and the protein expression of p62 decreased significantly. After SNHG1 expression was silenced and EZH2 was overexpressed, the protein expression of LC3-II and Beclin-1 in the tumor tissue was significantly reduced while the protein expression of p62 was significantly increased (**Figure 7E**). Compared with the NC group, the expression levels of EZH2, Wnt1, β-catenin, c-myc, and Cyclin D1 protein in the tumor tissue in the si-SNHG1 group were significantly reduced; the expression levels of EZH2, Wnt1, β-catenin, c-myc and Cyclin D1 protein were significantly increased in the tumor tissue in the si-SNHG1+EZH2 group (**Figure 7F**). Compared with the NC group, the expression levels of p-PI3K, p-AKT, p-mTOR, and p-p70S6K in the tumor tissue in the si-SNHG1 group were significantly reduced; the expression levels of p-PI3K, p-AKT, p-mTOR, and p-p70S6K in the tumor tissue in the si-SNHG1+EZH2 group were significantly increased (**Figure 7G**). It was confirmed that the results of *in vivo* and *in vitro* experiments were consistent.

## Discussion

It has been proved that many lncRNA play an important role in the occurrence and development of tumors. Abnormal expression of lncRNA has also been used as a prognostic marker for many tumors, such as lncRNA-AFAP1-AS1 (23), lncRNA-UCA1 (24), etc. Liu et al (16) found that the novel lnRNA-HOST2 can affect the transcription process upstream of the miR-let-7b promoter and significantly promote the expression of downstream proteins, thus enhancing the proliferation and migration of PCa cells. Other studies have pointed out that lncRNA-UCA1 can target and regulate zinc finger protein 703, increase its functional activity and enhance the proliferation ability of breast cancer cells (25). It is well-known that abnormal proliferation is one of the markers of tumor cells, and inhibiting the proliferation of tumor cells is the key to anti-tumor research (26, 27). Previous studies have found that downregulation of lncRNA-SNHG1 can inhibit the proliferation, colony formation and cell cycle transformation of gastric cancer cells (18). Downregulation of lncRNA-SNHG1 in lung cancer cells can not only inhibit the proliferation but also induce their apoptosis (28). Jiang et al. (20)found that SNHG1 increased the expression of CDK7 by competitively binding miR-199a-3p, thus promoting PCa cell proliferation and cell cycle progression. We would like to further study the effect of SNHG1 on the biological function and autophagy of PCa cells on the basis of this study, the high expression of SNHG1 in PCa tissues and PCa cells has been proved by RT-qPCR, which to some extent demonstrated the correlation between SNHG1 and the pathological process of PCa.

Polycarboxylate EZH2 is located on human chromosome 7q35 and contains 20 exons, nearly 40 kb in length, encoding 746 amino acids. The protein encoded by EZH2 contains a highly conserved SET domain, which has the activity of methyltransferase and inhibits the expression of downstream genes through methylation. EZH2 is a member of the PRC2 complex, and PRC2 is a highly conserved histone methyltransferase that acts on the lysine site k27 (H3k27) of histone h3. EZH2 is the core component of PRC2 and constitutes a catalytic subunit in the PRC2 protein complex (29). Recent study (30) showed that EZH2 is overexpressed in diverse human cancer tissues, such as HCC, breast cancer, PCa, lung cancer, gastric cancer, cervical cancer, lymphoma. Studies have found that EZH2 is closely related to tumor proliferation, metastasis and poor prognosis. The higher the expression level of EZH2, the higher the malignant degree of tumor and the worse the prognosis. For example, EZH2 expression in cervical cancer tissue is closely related to tumor proliferation and poor prognosis (31); degradation of EZH2 in breast cancer tissue weakens the invasion and metastasis of tumor (32). This study found that lncRNA-SNHG1 promoted the proliferation of PCa cells by up-regulating EZH2, that is, EZH2 activated by abnormal lncRNA-SNHG1 expression. It was subsequently confirmed that by interfering with SNHG1 or EZH2, the cell proliferation, colony formation, migration and invasion ability of PCa cells were significantly reduced, but the apoptosis and autophagy were significantly increased, while the opposite results occurred with interference with SNHG1 and overexpression of EZH2. These results have also been confirmed in *in vivo* animal experiments. This study suggests that SNHG1 inhibits the proliferation and metastasis of PCa cells by binding EZH2, and promotes the apoptosis and autophagy of cancer cells.

After determining the effect of SNHG1 on the biological characteristics of PCa cells, i the specific mechanism of this effect has become the focus of our next research. A large number of studies have confirmed that many signaling pathways (e.g. mTOR signaling pathway) play a key role in various cellular events, including regulation of gene expression, growth and proliferation. Studies have found that cell proliferation and metastasis are important causes of cancer occurrence and development. Wnt/β-catenin and PI3K/AKT signaling pathways are important signaling pathways that regulate cell proliferation and metastasis. They are often abnormally activated in the occurrence and development of many cancers including PCa, oral squamous cell carcinoma, skin squamous cell carcinoma (33–35). Wnt1, β-catenin, c-myc, CyclinD1 are key factors in Wnt/β-catenin signaling pathway (36), while PI3K and p-AKT are key molecules in PI3K/AKT signaling pathway (37). The increased expression of these factors is key to activate Wnt/β-catenin and PI3K/AKT signaling pathway (38). Furthermore, PI3K/AKT/mTOR pathway also plays a role in regulating cell autophagy. mTOR is a key regulator in the autophagy initiation phase (39). Activation of mTOR can inhibit cell autophagy (40, 41), leading to unlimited proliferation of tumor cells.

In this study, western blot was used to screen the correlation between SNHG1 and signal pathways. The results showed that interference with SNHG1 expression could lower the expression levels of wnt1, β-catenin, c-myc, CyclinD1, p-PI3K, p-AKT, p-mTOR, p-p70S6K. And simultaneous interference with SNHG1 and EZH2, the above gene expression was significantly up-regulated. This has also been confirmed by transfection of NC, si-SNHG1, si-SNHG1+Con, si-SNHG1+EZH2. in xenotransplantation experiments of tumor nude mice. Therefore, SNHG1 may activate Wnt/β-catenin and PI3K/AKT/mTOR signaling pathways.

In conclusion, SNHG1 is highly expressed in PCa cells and tissue. In-depth mechanism study confirmed that SNHG1 activates Wnt/β-catenin and PI3K/AKT/mTOR signaling pathways by targeting EZH2, and promoting proliferation, apoptosis and autophagy of PCa cells. According to the analysis of follow-up data, the expression of SNHG1 is positively correlated with the prognosis of PCa patients, while EZH2 is negatively correlated with the prognosis of PCa patients. It is suggested that SNHG1/EZH2 can be used as a prognostic marker for PCa, providing a new idea for early diagnosis and treatment of PCa. However, there is no clinical tissue specimens in this article. In the follow-up study, we will provide more reliable theoretical basis for SNHG1/EZH2 as a prognostic marker for PCa through clinical specimens.

## Data Availability Statement

The raw data supporting the conclusions of this article will be made available by the authors, without undue reservation.

## Ethics Statement

The studies involving human participants were reviewed and approved by Medical ethics committee of Second Affiliated Hospital of Fujian Medical University. Written informed consent from the patients/participants/legal guardian/next of kin was not required to participate in this study in accordance with the national legislation and the institutional requirements.

## Author Contributions

Study concept and design: JC and FW. Acquisition of data: HX, LX, and DC. Analysis and interpretation of data: JC and FW. Drafting of the manuscript: JC and FW. Critical revision of the manuscript for important intellectual content: JC and FW. Statistical analysis: JW and SH. Administrative, technical, and material support: YW and LF. Study supervision: JC and FW. All authors contributed to the article and approved the submitted version.

## Funding

Quanzhou Science and Technology Project (2018C048R).

## Conflict of Interest

The authors declare that the research was conducted in the absence of any commercial or financial relationships that could be construed as a potential conflict of interest.

## References

[1] Helzlsouer KJ Huang HY Alberg AJ Hoffman S Burke A Norkus EP Association Between α-Tocopherol, γ-Tocopherol, Selenium, and Subsequent Prostate Cancer. J Natl Cancer Inst (2018) 92(24):2018–23. 10.1093/jnci/92.24.2018 11121464

[2] Mikolajczyk SD Catalona WJ Evans CL Linton HJ Millar LS Marker KM Proenzyme Forms of Prostate-Specific Antigen in Serum Improve the Detection of Prostate Cancer. Clin Chem (2004) 50(6):1017–25. 10.1373/clinchem.2003.026823 15054080

[3] Heinlein CA Chang C Androgen Receptor in Prostate Cancer. Endoc Rev (2004) 25(2):276–308. 10.1210/er.2002-0032 15082523

[4] Mohler J Babaian RJ Bahnson RR Boston B D’Amico A Eastham JA Prostate cancer. Clinical practice guidelines in oncology. J Urol (2015) 193(4):1153–8. 10.1016/j.juro.2014.10.105

[5] Moyer VA U.S. Preventive Services Task Force Screening for Prostate Cancer: U.S. Preventive Services Task Force Recommendation Statement. Ann Internal Med (2012) 157(2):120–34. 10.7326/0003-4819-157-2-201207170-00459 22801674

[6] Scher H II Fizazi K Saad F Taplin ME Sternberg CN Miller K Increased Survival with Enzalutamide in Prostate Cancer after Chemotherapy. N Engl J Med (2012) 367(13):1187–97. 10.1056/NEJMoa1207506 22894553

[7] Spizzo R Almeida M II Colombatti A Calin GA Long non-coding RNAs and cancer: a new frontier of translational research? Oncogene (2012) 31(43):4577–87. 10.1038/onc.2011.621 PMC3433647 22266873

[8] Wang KC Chang HY Molecular mechanisms of long noncoding RNAs. Mol Cell (2011) 43(6):904–14. 10.1016/j.molcel.2011.08.018 PMC3199020 21925379

[9] Kopp F Mendell JT Functional Classification and Experimental Dissection of Long Noncoding RNAs. Cell (2018) 172(3):393–407. 10.1016/j.cell.2018.01.011 29373828 PMC5978744

[10] Sarfi M Abbastabar M Khalili E Long noncoding RNAs biomarker-based cancer assessment. J Cell Physiol (2019) 234 (10):16971–86. 10.1002/jcp.28417 30835829

[11] Camacho CV Choudhari R Gadad SS Long Noncoding RNAs and Cancer, an Overview. Steroids (2018) 133:93–5. 10.1016/j.steroids.2017.12.012 29317255

[12] Ye ZQ Wang T Song W [Long noncoding RNAs in prostate cancer]. Zhonghua Nan Ke Xue (2014) 20(11):963–8. 25577829

[13] Shi X Zhang W Nian X The previously uncharacterized lncRNA APP promotes prostate cancer progression by acting as a competing endogenous RNA. Int J Cancer (2020) 146(2):475–86. 10.1002/ijc.32422 31107971

[14] Li H Liu C Lu Z Lu X Li Y Liu F Upregulation of the long non-coding RNA SPRY4-IT1 indicates a poor prognosis and promotes tumorigenesis in ovarian cancer. Biomed Pharmacother (2017) 88:529–34. 10.1016/j.biopha.2017.01.037 28129625

[15] Chakravarty D Sboner A Nair SS Giannopoulou E Li R Hennig S The oestrogen receptor alpha-regulated lncRNA NEAT1 is a critical modulator of prostate cancer. Nat Commun (2014) 5:5383. 10.1038/ncomms6383 25415230 PMC4241506

[16] Liu RT Cao JL Yan CQ Wang Y An CJ Lv HT Effects of LncRNA-HOST2 on cell proliferation, migration, invasion and apoptosis of human hepatocellular carcinoma cell line SMMC-7721. Biosci Rep (2017) 37(2):BSR20160532. 10.1042/BSR20160532 28143959 PMC5398253

[17] Zhang H Zhou D Ying M Chen M Chen P Chen Z Expression of long non-coding RNA (lncRNA) small nucleolar RNA host gene 1 (SNHG1) exacerbates hepatocellular carcinoma through suppressing miR-195. Med Sci Monit (2016) 22:4820–29. 10.12659/MSM.898574 PMC5167104 27932778

[18] Hu Y Ma Z He Y Liu W Su Y Tang Z LncRNA-SNHG1 contributes to gastric cancer cell proliferation by regulating DNMT1. Biochem Biophys Res Commun (2017) 491(4):926–31. 10.1016/j.bbrc.2017.07.137 28754593

[19] Yan Y Fan Q Wang L Zhou Y Li J Zhou K LncRNA Snhg1, a non-degradable sponge for miR-338, promotes expression of proto-oncogene CST3 in primary esophageal cancer cells. Oncotarget (2017) 8(22):35750–60. 10.18632/oncotarget.16189 PMC5482614 28423738

[20] Li J Zhang Z Xiong L Guo C Jiang T Zeng L SNHG1 lncRNA negatively regulates miR-199a-3p to enhance CDK7 expression and promote cell proliferation in prostate cancer. Biochem Biophys Res Commun (2017) 487(1):146–52. 10.1016/j.bbrc.2017.03.169 28400279

[21] Yun C Zhang F Zhu C Geng L Tian T Tian H Upregulated lncRNA SNHG1 contributes to progression of non-small cell lung cancer through inhibition of miR-101-3p and activation of Wnt/β-catenin signaling pathway. Oncotarget (2017) 8(11):17785–94. 10.18632/oncotarget.14854 PMC5392286 28147312

[22] Xu M Chen X Lin K Zeng K Liu X Pan B The long noncoding RNA SNHG1 regulates colorectal cancer cell growth through interactions with EZH2 and miR-154-5p. Mol Cancer (2018) 17(1):141. 10.1186/s12943-018-0894-x 30266084 PMC6162892

[23] Wang F Ni H Sun F Li M Chen L Overexpression of lncRNA AFAP1-AS1 correlates with poor prognosis and promotes tumorigenesis in colorectal cancer. Biomed Pharmacother (2016) 81:152–9. 10.1016/j.biopha.2016.04.009 27261589

[24] Nie W Ge H-J Yang X-Q Sun X Huang H Tao X LncRNA-UCA1 exerts oncogenic functions in non-small cell lung cancer by targeting miR-193a-3p. Cancer Lett (2016) 371(1):99–106. 10.1016/j.canlet.2015.11.024 26655272

[25] Tan W Song Z-Z Xu Q Qu X Li Z Wang Y Up-regulated expression of SPRY4-IT1 predicts poor prognosis in colorectal cancer. Med Sci Monit: Int Med J Exp Clin Res (2017) 23:309–14. 10.12659/MSM.898369 PMC5267619 28099409

[26] Zhu Z-M Liu F-T Chen X Low expression of LncRNA cancer susceptibility candidate 2 and its clinical significance in cancer tissues. Cell Physiol Biochem (2018) 46(4):1643–9. 10.1159/000489211 29694965

[27] Yang G Lu X Yuan L LncRNA: a link between RNA and cancer. Biochim Biophys Acta (BBA)-Gene Regul Mech (2014) 1839(11):1097–109. 10.1016/j.bbagrm.2014.08.012 25159663

[28] Cui Y Zhang F Zhu C Geng L Tian T Liu H Upregulated lncRNA SNHG1 contributes to progression of non-small cell lung cancer through inhibition of miR-101-3p and activation of Wnt/β-catenin signaling pathway. Oncotarget (2017) 8(11):17785. 10.18632/oncotarget.14854 28147312 PMC5392286

[29] Jiang T Wang Y Zhou F Gao G Ren S Zhou C Prognostic value of high EZH2 expression in patients with different types of cancer: a systematic review with meta-analysis. Oncotarget (2016) 7(4):4584–97. 10.18632/oncotarget.6612 PMC4826228 26683709

[30] Schmitt AM Chang HY Long Noncoding RNAs in Cancer Pathways. Cancer Cell (2016) 29(4):452–63. 10.1016/j.ccell.2016.03.010 PMC4831138 27070700

[31] Li Z Hou P Fan D Dong M Ma M Li H The degradation of EZH2 mediated by lncRNA ANCR attenuated the invasion and metastasis of breast cancer. Cell Death Differ (2017) 24(1):59–71. 10.1038/cdd.2016.95 27716745 PMC5260507

[32] Italiano A Role of the EZH2 histone methyltransferase as a therapeutic target in cancer. Pharmacol Ther (2016) 165:26–31. 10.1016/j.pharmthera.2016.05.003 27179746

[33] Pak S Park S Kim Y Park J-H Park C-H Lee K-J The small molecule WNT/β-catenin inhibitor CWP232291 blocks the growth of castration-resistant prostate cancer by activating the endoplasmic reticulum stress pathway. J Exp Clin Cancer Res (2019) 38(1):342. 10.1186/s13046-019-1451-1 31387608 PMC6685284

[34] Sang HL Koo BS Kim JM Huang S Rho YS Bae WJ Wnt/β-catenin signaling maintains self-renewal and tumorigenicity of head and neck squamous cell carcinoma stem-like cells by activating Oct4. J Pathol (2014) 234(1):99–107. 10.1002/path.4383 24871033

[35] Wu N Du Z Zhu Y Song Y Pang L Chen Z The Expression and Prognostic Impact of the PI3K/AKT/mTOR Signaling Pathway in Advanced Esophageal Squamous Cell Carcinoma. Technol Cancer Res Treat (2018) 17. 10.1177/1533033818758772 PMC5826005 29463194

[36] Yoshida T Sopko NA Kates M Liu X Joice G McConkey DJ Wnt/β-catenin pathway in proliferation of bladder cancer cells. Oncotarget (2018) 9(13):11060–70. 10.18632/oncotarget.24308 PMC5834271 29541396

[37] Díaz-Serrano A Angulo B Dominguez C Pazo-Cid R Salud A Jiménez-Fonseca P Genomic Profiling of HER2-Positive Gastric Cancer: PI3K/Akt/mTOR Pathway as Predictor of Outcomes in HER2-Positive Advanced Gastric Cancer Treated with Trastuzumab. Oncologist (2018) 23(9):1092–102. 10.1634/theoncologist.2017-0379 PMC6192610 29700210

[38] Deng F Liang P Li Z Tan G Liang E Chen S YAP triggers the Wnt/β-catenin signalling pathway and promotes enterocyte self-renewal, regeneration and tumorigenesis after DSS-induced injury. Cell Death Dis (2018) 9(2):153. 10.1038/s41419-017-0244-8 29396428 PMC5833613

[39] Wu Z You Z Chen P Chen C Chen F Shen J Matrine Exerts Antidepressant-Like Effects on Mice: Role of the Hippocampal PI3K/Akt/mTOR Signaling. Int J Neuropsychopharmacol (2018) 21(8):764–76. 10.1093/ijnp/pyy028 PMC6070064 29668939

[40] Kang EB Cho JY Effect of treadmill exercise on PI3K/AKT/mTOR, autophagy, and Tau hyperphosphorylation in the cerebral cortex of NSE/htau23 transgenic mice. J Exerc Nutrition Biochem (2015) 19(3):199–209. 10.5717/jenb.2015.15090806 PMC4624121 26527331

[41] Yin S Yang S Pan X Ma A Ma J Pei H MicroRNA-155 promotes ox-LDL-induced autophagy in human umbilical vein endothelial cells by targeting the PI3K/Akt/mTOR pathway. Mol Med Rep (2018) 18(3):2798–806. 10.3892/mmr.2018.9236 PMC6102700 30015881

