# Corrigendum: Long non-coding RNA SNHG1 regulates the Wnt/β-catenin and PI3K/AKT/mTOR signaling pathways *via* EZH2 to affect the proliferation, apoptosis, and autophagy of prostate cancer cell

**DOI:** 10.3389/fonc.2024.1501882

**Published:** 2024-11-25

**Authors:** Junyi Chen, Fubo Wang, Huan Xu, Lingfan Xu, Dong Chen, Jialiang Wang, Sihuai Huang, Yiqun Wen, Longmin Fang

**Affiliations:** ^1^ Department of Urology, The Second Affiliated Hospital of Fujian Medical University, Quanzhou, China; ^2^ Department of Urology, Shanghai Changhai Hospital, Naval Medical University, Shanghai, China; ^3^ Department of Urology, The First Affiliated Hospital of Anhui Medical University, Hefei, China

**Keywords:** prostate cancer, long-chain non-coding RNA, cell viability, apoptosis, autophagy

In the published article, there was an error in **Figures 4**, **5** as published. Due to the failure to replace the latest figures during repair and the proof not being thoroughly checked, errors were found in **Figures 4**, **5**. The corrected **Figures 4**, **5** and its caption appear below.

The authors apologize for this error and state that this does not change the scientific conclusions of the article in any way. The original article has been updated.

**Figure 4:**
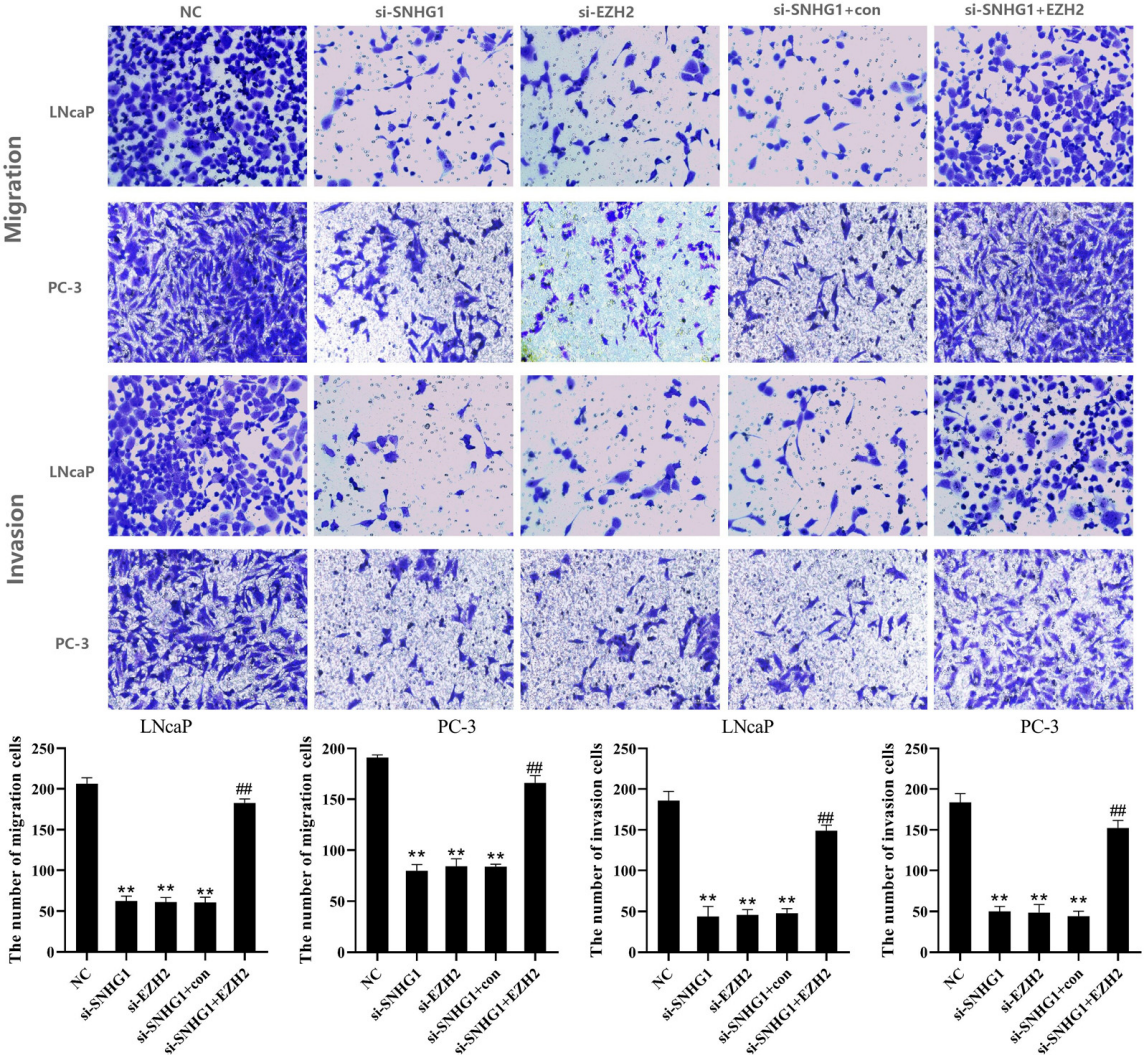
Regulatory effect of SNHG1 on the migration and invasion of PCa cells by targeting EZH2. Transwell assay detected thel migration and invasion of LNCaP and PC3 cells transfected with NC, si-SNHG1, si-EZH2, si-SNHG1+con, si-SNHG1+EZH2. **P < 0.01 vs. NC group; ^##^P < 0.01 vs. si-SNHG1 + con group.

**Figure 5:**
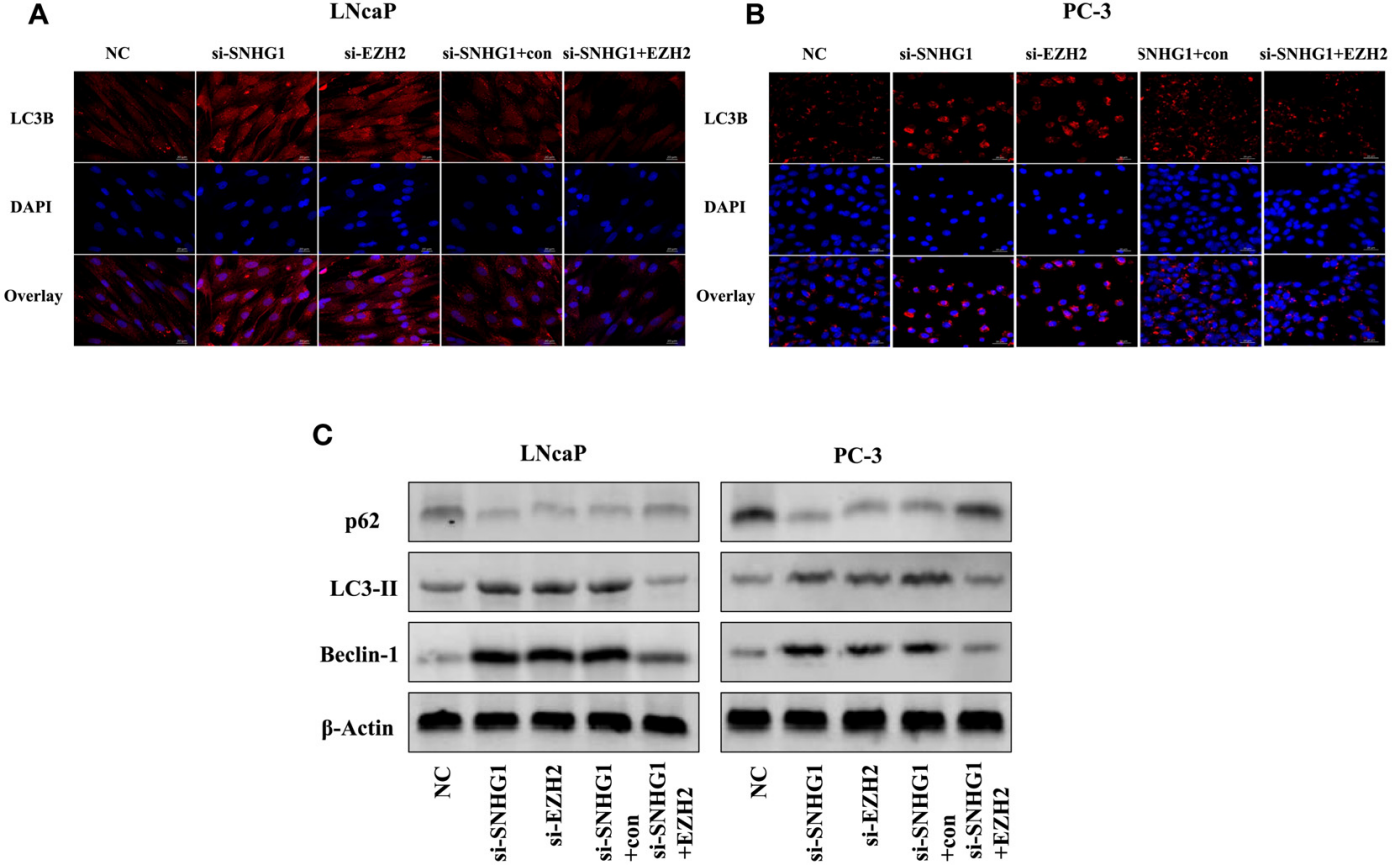
Effect of SNHG1 binding EZH2 on autophagy in LNCap and PC-3 cells. **(A, B)** Immunofluorescence staining detected the formation of the LC3 spots with the interference of SNHG1 in LNCaP and PC3 cells and the down-regulation of EZH2 or the interference of SNHG1 and simultaneous overexpression of EZH2. **(C)** Western blot detected the expression level of autophagy-related proteins LC3-II, Beclin-1 and P62 protein in LNCaP and PC3 cells transfected with NC, si-SNHG1, si-EZH2, si-SNHG1+con, si-SNHG1+EZH2.

